# Blocking drug activation as a therapeutic strategy to attenuate acute toxicity and physiological effects of heroin

**DOI:** 10.1038/s41598-018-35196-8

**Published:** 2018-11-13

**Authors:** Ting Zhang, Xirong Zheng, Kyungbo Kim, Fang Zheng, Chang-Guo Zhan

**Affiliations:** 1 Molecular Modeling and Biopharmaceutical Center, College of Pharmacy, University of Kentucky, 789 South Limestone Street, Lexington, KY 40536 USA; 2 Department of Pharmaceutical Sciences, College of Pharmacy, University of Kentucky, 789 South Limestone Street, Lexington, KY 40536 USA

## Abstract

Heroin is a growing national crisis in America. There is an increasing frequency of heroin overdoses. All of the currently used therapeutic approaches to treatment of heroin abuse and other opioid drugs of abuse focus on antagonizing a brain receptor (particularly µ-opiate receptors). However, it has been known that the therapeutic use of certain µ-opiate receptor antagonist may actually increase heroin overdose. Once overdosed, heroin addicts may continue to get overdosed again and again until fatal. Here we report our design and validation of a novel therapeutic strategy targeting heroin activation based on our analysis of the chemical transformation and functional change of heroin in the body. An effective blocker of heroin activation, such as ethopropazine tested in this study, may be used as a standalone therapy or in combination with a currently available, traditional medications targeting µ-opiate receptors (*e*.*g*. naltrexone or its extended-release formulation Vivitrol). The combination therapy would be ideal for heroin abuse treatment as the effects of two therapeutic agents targeting two independent mechanisms are cooperative.

## Introduction

Heroin is known as a growing national crisis in America, due to the rapidly increasing overdose deaths. From 2001 to 2014, heroin overdose deaths nationwide increased 594 percent and continued to dramatically increase since 2014, according to the Centers for Disease Control (https://www.cdc.gov/drugoverdose/epidemic/index.html), and “*heroin is scarring the next generation*”^1^. The connection between heroin abuse and prescription opioid abuse is related to the actual availability, in addition to the common brain protein target (µ-opiate receptors), of these opioid drugs. In fact, “*80% of recent heroin initiates reported that they began their opioid use through the nonmedical use of prescription opioid medications*”^2^. The prevalence of prescription opioid abuse is similar among men and women. Those who abuse the prescription drugs most often obtain them from friends and family either through sharing or theft. When they are no longer able to get prescription opioid drugs, they start to use illegal opioid heroin, because heroin is easy to obtain on the street and even online. In addition, heroin has become much cheaper than any other drug of abuse, *e*.*g*. $10–$20 for a typical single dose (0.1 g) of heroin purchased on the street^2^. Due to the connection between heroin abuse and prescription opioid abuse, further enhancing law enforcement through improving existing prescription drug monitoring programs^3^ might effectively reduce the prescription opioid abuse. On the flip side, the reduced prescription opioid abuse could lead to increase of the heroin abuse. As a result, the overall opioid overdose deaths might not really decrease. For example, study showed prescription drug abuse declining in Kentucky^4^, due to the strict Kentucky All Schedule Prescription Electronic Reporting Program (KASPER). Unfortunately, the heroin overdose has dramatically increased^4^, and the total number of drug overdose deaths actually went up in Kentucky^2,5^. So, further enhancing law enforcement through improving prescription drug monitoring programs should be associated with development of a more effective therapeutic strategy for heroin abuse.

Currently used therapeutic agents for treatment of heroin abuse and other opioid drugs of abuse include naloxone for overdose treatment and buprenorphine, methadone, and naltrexone for addiction treatment. These therapeutic agents may be used in various formulations/devices, such as nasal spray device for naloxone^6^ for fast overdose treatment and extended-release naltrexone for relapse-preventing addiction treatment^7^. All of these therapeutic agents in the current clinical use, and other therapeutic candidates under preclinical/clinical development, bind to µ-opiate receptors (and/or a related receptors) in the brain and, thus, block the physiological effects of heroin or another opioid used. The overdose treatment with naloxone appears to be effective, but the precondition is that the naloxone treatment can begin soon enough after a heroin overdose. For the problem, once overdosed, heroin addicts may continue to get overdosed again and again until fatal. Some heroin addicts survived from one overdose with treatment in hospital, and then died of another overdose the next day^2^. Even worse, *the use of naltrexone or its extended-release formulation Vivitrol actually increased heroin overdose*^8–10^. A truly effective heroin treatment should account for not only rescuing heroin addicts who have already been overdosed, but also preventing the addicts from overdose again.

Here we report our mechanism-based rational design and validation of a novel therapeutic strategy targeting heroin activation to complement the currently available antagonist approach. Our strategy is based on the fact that heroin itself is actually a prodrug which must be activated to be effective, and our further analysis of the chemical transformation and functional change of heroin in the body. An effective blocker of heroin activation may be used as a standalone therapy or in combination with one of the currently available traditional medications targeting µ-opiate receptors (*e*.*g*. naltrexone or its extended-release formulation Vivitrol). The combination therapy would be ideal for heroin abuse treatment as the effects of two therapeutic agents targeting two independent mechanisms are perfectly cooperative.

## Results

### Catalytic parameters of the primary enzyme involved in heroin activation

**Figure 1:**
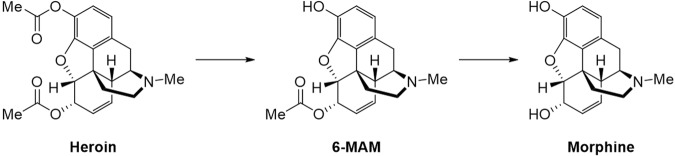
The metabolic pathway of heroin to morphine – hydrolysis reactions catalyzed by cholinesterases.

It has been known that heroin (3,6-diacetylmorphine, synthesized from morphine) behaves as a prodrug and its effects are mediated by its metabolites (6-MAM and morphine depicted in Fig. 1)^11,12^. Once administered, heroin is very rapidly metabolized by cholinesterases to 6-monoacetylmorphine (6-MAM) first, and then to morphine (see Fig. 1). Both 6-MAM and morphine are responsible for the toxic and physiological effects of heroin^13–20^. In fact, heroin is at least 10-fold more toxic than morphine^16^, but the binding affinity of heroin itself with the µ-opiate receptors is significantly lower than that of morphine. The most toxic metabolite is 6-monoacetylmorphine (6-MAM) which has the highest binding affinity with the µ-opiate receptors. 6-MAM (with a relatively shorter biological half-life compared to morphine) is mainly responsible for the acute toxicity^17^, whereas morphine (with a much longer biological half-life) is mainly responsible for the long-term toxicity, of heroin. So, cholinesterases are crucial enzymes that control the metabolic profile, and thus affect the toxic and physiological effects, of heroin.

**Figure 2:**
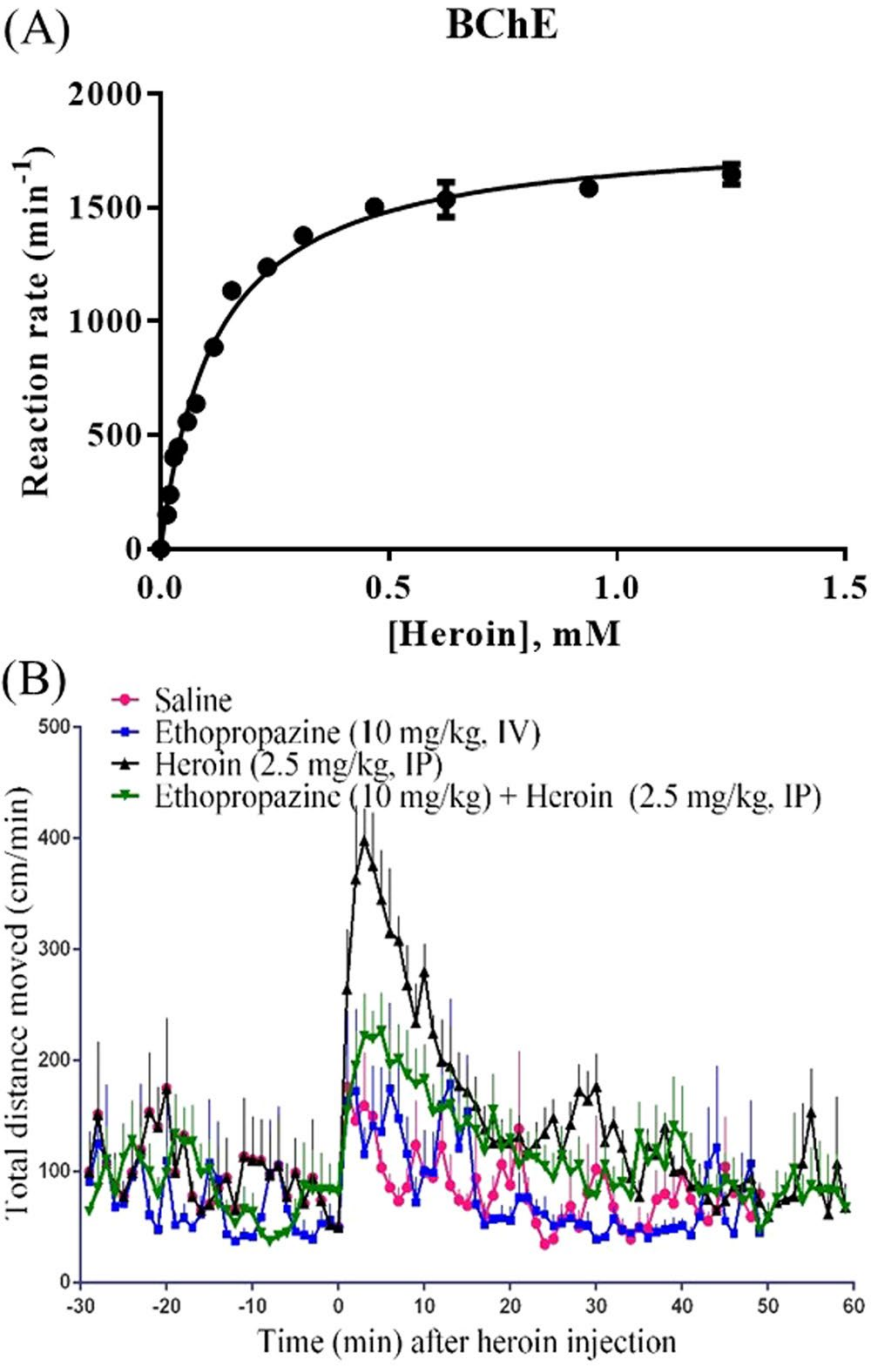
(**A**) Plot of measured initial reaction rates (represented in nM min^−1^ per nM enzyme, or min^−1^, at 37 °C, measured in triplicate, with error bars in standard deviations) *versus* the substrate (heroin) concentration for recombinant human BChE-catalyzed hydrolysis of heroin. (**B**) Locomotor activity in mice expressed as total distance traveled (in centimeters) per min after heroin injection (2.5 mg/kg, IP) or saline, with or without ethopropazine injection (10 mg/kg, IV) 10 min prior to the heroin (or nothing) injection (2.5 mg/kg, IP) at the time 0. A group of eight mice (n = 8) were used at the same time for each dose condition.

There are two main cholinesterases, including acetylcholinesterase (AChE) and butyrylcholinesterase (BChE). Which cholinesterase (AChE or BChE) is mainly responsible for heroin hydrolysis has been controversial. It was once proposed that human erythrocyte AChE is responsible for hydrolyzing heroin^21^. However, further studies by Lockridge *et al*. indicated that human serum BChE, instead of erythrocyte AChE, is responsible for hydrolyzing heroin^13^. As previously reported catalytic parameters for BChE-catalyzed hydrolysis of heroin were inconsistent (*k*_cat_ = 12.5 to 540 min^−1^ and *K*_M_ = 110 to 3500 µM)^13,22,23^, we performed the Michaelis-Menten kinetic analysis of the BChE-catalyzed hydrolysis of heroin at 37 °C. The kinetic data (Fig. 2A) reveal that recombinant human BChE is highly efficient for heroin hydrolysis (*k*_cat_ = 1840 min^−1^ and *K*_M_ = 120 µM), which is qualitatively consistent with the conclusion obtained by Lockridge *et al*.^13^. According to reported pharmacokinetic studies^17^, heroin is converted to 6-MAM with a very high metabolic rate in blood (with a rate constant for heroin hydrolysis to 6-MAM as high as 12.092 min^−1^), and the estimated metabolic rate of heroin in brain was much lower. The very different metabolic rates for heroin in blood and brain calculated by the model were confirmed by *in vitro* experiments^18^, which also supports the notion that BChE (which is the dominant cholinesterase in serum) is the primary enzyme responsible for heroin activation. Further, heroin is either eliminated very rapidly from the body (by some direct pathway, *e*.*g*. urine) or metabolized rapidly to 6-MAM in plasma, according to the well-established pharmacokinetic (PK) model (the rate constant is 13.757 min^−1^ for direct heroin elimination and 12.092 min^−1^ for heroin hydrolysis to 6-MAM)^18^. Therefore, we propose to target heroin activation by using a BChE-selective inhibitor in order to attenuate the toxic and physiological effects of heroin. When the metabolic pathway of heroin by BChE is blocked, heroin will mainly go through the direct elimination process to urine. Hence, blocking BChE-catalyzed hydrolysis of heroin should help to significantly attenuate the actual toxicity and physiological effects of heroin.

### Effects of ethopropazine on heroin-induced hyperactivity

Notably, many cholinesterase inhibitors have been approved by the FDA as therapeutic agents for treatment of Alzheimer’s disease (AD), myasthenia gravis, glaucoma, and Parkinson’s disease *etc*. Most of these cholinesterase inhibitors are able to inhibit both AChE and BChE, without the desirable high selectivity for BChE over AChE. Nevertheless, ethopropazine (a BChE inhibitor approved by the FDA for treatment of Parkinson’s disease) is highly selective for BChE over AChE, with IC_50_ = 210–300 nM for BChE and IC_50_ = 210 μM for AChE (~1000-fold selectivity)^24^. So, ethopropazine was used to examine whether a BChE-selective inhibitor can really attenuate the toxic and physiological effects of heroin in mice. First, we determined the effects of ethopropazine on heroin-induced hyperactivity by performing locomotor activity tests in high density, non-porous plastic chambers measuring 50 cm (L) × 50 cm (W) × 38 cm (H) in a light- and sound-attenuating behavioral test enclosure (San Diego Instruments, San Diego, CA). As seen in Fig. 2, ethopropazine (10 mg/kg, IV) administration 10 min prior to the heroin (2.5 mg/kg, IP) injection significantly attenuated heroin-induced hyperactivity.

### Effects of ethopropazine on heroin-induced acute toxicity

**Figure 3:**
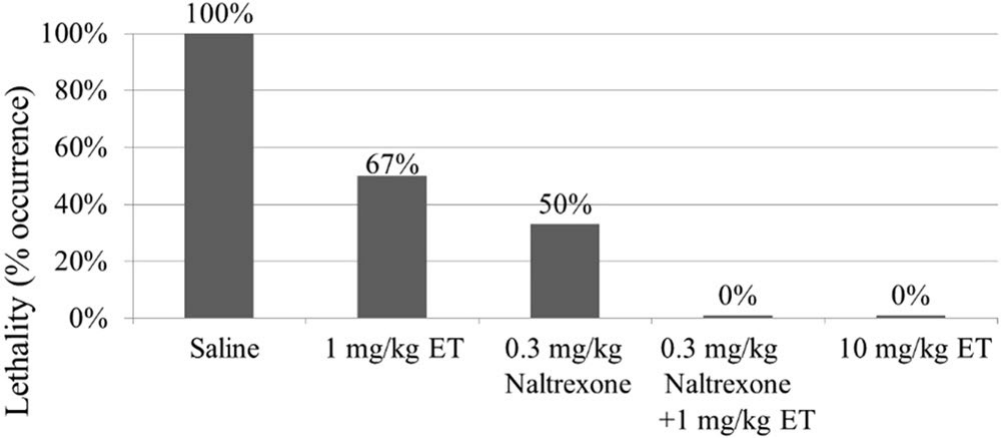
Effectiveness of ethopropazine (ET) and naltrexone in protection of mice against the acute toxicity of a lethal dose of heroin (25 mg/kg, IV). ET and/or naltrexone (IV) was administered (IV) 10 min before the heroin administration, in comparison with the control group (saline) without ET or naltrexone administration. Each data point represents the percentage of mice (n = 6 for each dose condition) exhibiting heroin-induced lethality.

Ethopropazine was tested further for its effectiveness in protecting mice against heroin-induced acute toxicity. Heroin-induced acute toxicity was characterized by the lethality (occurrence of death). For the protection experiments, a single dose of ethopropazine was administrated (IV) prior to administration of a lethal dose (LD_100_) of heroin (*e*.*g*. 25 mg/kg, IV). At 10 min after IV administration of ethopropazine or saline (negative control), the mice were given a lethal dose (LD_100_) of heroin. The protection experiments were performed under various dose conditions (n = 6 for each scenario). Depicted in Fig. 3 are the data obtained from the protection experiments with ethopropazine (1 or 10 mg/kg, IV) and/or naltrexone (0.3 mg/kg, IV); we also tried to use a higher dose (1 mg/kg, IV) of naltrexone and noted that the IV dose of 1 mg/kg would be too high because the mice injected intravenously with 1 mg/kg naltrexone were not able to stand and could hardly move at all.

The data depicted in Fig. 3 reveal that, without ethopropazine or naltrexone administration, all mice died within 2 min after the heroin injection (25 mg/kg, IV). However, pre-treatment of mice with ethopropazine (10 mg/kg, IV) 10 min before the heroin injection protected all mice against the acute toxicity of a lethal dose of heroin (25 mg/kg, IV). So, 10 mg/kg ethopropazine was able to protect all of the mice from the heroin-induced lethality (full protection).

Further, we also tested whether Galantamine (a highly potent and selective inhibitor of AChE, approved by the FDA for treatment of Alzheimer’s disease) can also attenuate heroin-induced toxicity, and found that pre-treatment of mice with Galantamine at a dose of 10 or 5 mg/kg (IV or IP) before the heroin injection did not protect any mouse against the acute toxicity of a lethal dose of heroin (25 mg/kg, IV), which is consistent with the concept that BChE, rather than AChE, is the primary heroin-activating enzyme.

Additional data in Fig. 3 indicate that 1 mg/kg ethopropazine or 0.3 mg/kg naltrexone only provided partial protection, but the combined use of 0.3 mg/kg naltrexone and 1 mg/kg ethopropazine provided the full protection. So, the protection effects of ethopropazine and naltrexone are indeed cooperative, as expected.

## Discussion

All of the animal data obtained consistently support the concept of using a potent BChE-selective inhibitor, such as ethopropazine tested in this study, as a blocker of heroin activation to attenuate the toxicity and physiological effects of heroin. For this reason, ethopropazine may be repurposed for clinical use to attenuate the acute toxicity of heroin.

Further, although a heroin activation blocker, such as ethopropazine which we have identified in this study for the first time, may be used as a standalone therapy for heroin addiction treatment, the combined use of a heroin activation blocker and a μ-opiate receptor antagonist should be more effective. This is because the two mechanisms are independent and, therefore, their effects can be perfectly cooperative (Bliss Independence model)^25,26^. The cooperative effects of the two therapeutic agents should be much more effective. For example, if a heroin activation blocker with a given concentration in plasma can block heroin activation by 90% and if the μ-opiate receptor antagonist with a given concentration in the brain can block the receptors binding with the available active heroin metabolites (associated with 10% of the total heroin) also by 90%, then only 10% of the 10% (*i*.*e*. 1% of the total) can bind with μ-opiate receptors. Thus, the two types of therapeutic agents together can block the toxicity and physiological effects of heroin by 99%, when each one can block the toxicity and physiological effects of heroin by 90%. For the same logics, the two types of therapeutic agents together can block the toxicity and physiological effects of heroin by 75%, when each one can block them by 50%; the two types of therapeutic agents together can block the toxicity and physiological effects of heroin by 99.99%, when each one can block them by 99%. So, the novel combination therapy is potentially more promising for heroin addiction treatment and for prevention of heroin overdose. In addition, the general concept of a combination therapy with two therapeutic agents (*e*.*g*. ethopropazine combined with naltrexone or its extended-release formulation Vivitrol) targeting two independent mechanisms may also be useful for therapeutic treatment of other drugs of abuse.

## Materials and Methods

### Materials

The purified recombinant human BChE protein was prepared in our previous study^27^. Briefly, the BChE protein was expressed in CHO-S cells, and the secreted enzyme in the culture medium was purified by a two-step approach, including ion exchange chromatography using QFF anion exchanger and affinity chromatography using procainamide-sepharose. The purified protein was stored at −80 °C before the use. Heroin and its metabolite 6-MAM were provided by the National Institute on Drug Abuse (NIDA) Drug Supply Program (Bethesda, MD). All other supplies (including ethopropazine and naltrexone) were purchased from Sigma-Aldrich (St. Louis, MO).

### Animals

Male CD-1 mice (28–32 g) were ordered from Harlan (Harlan, Indianapolis, IN), and housed in cage. All mice were allowed ad libitum access to food and water and maintained on a 12 h light/12 h dark cycle, with the lights on at 8:00 a.m. at a room temperature of 21–22 ^o^C. Experiments were performed in a same colony room in accordance with the Guide for the Care and Use of Laboratory Animals as adopted and promulgated by the National Institutes of Health. The animal protocol was approved by the IACUC (Institutional Animal Care and Use Committee) at the University of Kentucky.

### *In vitro* enzyme activity assay

For kinetic analysis of the catalytic activity of BChE against heroin, the purified enzyme and heroin were incubated in 0.1 M phosphate buffer, pH 7.4, at 37 °C. The enzymatic reaction was terminated and the protein was precipitated by adding 100 µl of iced 50% acetonitrile/0.5 M hydrochloric acid, followed by 5 min centrifugation at 15,000 *g*. The resulting supernatant was subjected to reverse-phase HPLC (RP-HPLC) on a 5 µm C18 110 Å column (250 × 4.6 mm; Gemini) and RP-HPLC was performed using the mobile phase consisting of 20% acetonitrile in 0.1% TFA. The remaining substrate (heroin) and the formed reaction product (6-MAM) were monitored by a fluorescence detector with an excitation wavelength of 230 nm and emission wavelength of 315 nm and by monitoring UV absorbance at 230 nm. The quantification was based on a standard curve prepared using an authentic standard compound. The measurement was performed in triplicate, and the catalytic parameters were determined by using the Michaelis-Menten kinetic equation.

### Locomotor activity assay

To determine the effects of ethopropazine on heroin-induced hyperactivity, locomotor activity tests were performed in high density, non-porous plastic chambers measuring 50 cm (L) × 50 cm (W) × 38 cm (H) in a light- and sound-attenuating behavioral test enclosure (San Diego Instruments, San Diego, CA). This system available in our lab can test 8 mice at the same time. Cumulative distance traveled and speed were recorded by EthoVision XT video tracking system (Noldus Information Technology, Wageningen, Netherlands) to represent the locomotor activity. Mice were introduced to the test chambers for one habituation session (no injection). Before the test session, mice were allowed to acclimate to the test chambers for at least 60 minutes, and the total distance traveled during this period of time were determine the basal activity. Then, ethopropazine or saline (negative control) was administered through intravenous (IV) injection. At 10 min after the ethopropazine or saline injection, the mice were given a pharmacological dose of heroin (*e*.*g*., 2.5 mg/kg, IP). After the heroin administration, mice were immediately returned to the test chamber for a 60-minute session of activity monitoring.

### Protection study in mice

Heroin-induced acute toxicity was characterized in this study by the occurrence of death. Protection experiment was performed by pretreatment of mice with ethopropazine (1 or 10 mg/kg, IV) and/or naltrexone (0.3 mg/kg) 10 min before administration of heroin (25 mg/kg, IV). Following the heroin administration, mice were immediately placed in containers for observation. The presence or absence of death was recorded for 6 hours following heroin administration.
